# A two-factor scale of perceived power

**DOI:** 10.1371/journal.pone.0319412

**Published:** 2025-02-28

**Authors:** Myojoong Kim, Frank May

**Affiliations:** 1 Independent Researcher, Ulsan, South Korea; 2 Department of Marketing, Virginia Tech, Blacksburg, Virginia, United States of America; SGH Warsaw School of Economics: Szkola Glowna Handlowa w Warszawie, POLAND

## Abstract

Power-the capacity to influence outcomes-manifests in two distinct forms: social power, defined as the perceived ability to control others’ behaviors and decisions, and personal power, characterized by the capacity to resist unwanted external influence and maintain autonomy. Theoretically, these dimensions are rooted in different needs, and thus are likely to differentially predict certain behaviors. However, existing measures often conflate these dimensions, limiting insights into their unique behavioral effects. To address this issue, the present research has developed and validated a two-factor scale of perceived power to completely capture both facets of power across twelve studies (N =  2,878). Exploratory and confirmatory factor analyses support the scale’s structure, while reliability and validity tests demonstrate its robustness. Following assessments of the structure of the scale, its validity was demonstrated across multiple studies: Study 1 establishes the orthogonality of personal and social power through experimental manipulation, Study 2 reveals that personal power increases proactive advice-seeking, whereas social power reduces the tendency to solicit advice, and Study 3 demonstrates that social power amplifies negative reactions to service failures, while personal power does not. These divergent outcomes underscore the distinct roles of personal and social power, highlighting the scale’s utility for advancing research.

## Introduction

Power has garnered considerable attention across various disciplines such as marketing [1–4], management [5,6], and psychology [7–12]. Power significantly influences various behaviors, including compensatory consumption [13], risk-taking behavior [2], and switching behavior [14], highlighting its relevance across various fields.

Traditionally, power research has focused on social power, defined as the ability to influence and persuade others [9,15,16]. While this aspect is well-documented, studies have also highlighted the importance of personal power - the capacity to resist external influences and control one’s own outcomes [15,17,19].

Despite the recognition of personal power, existing research often conflates it with social power or overlooks it entirely [18]. Many studies employ measures that emphasize social power, potentially neglecting individuals’ ability to resist others’ influence [19,20]. Furthermore, power manipulations in research often conflate social and personal power, potentially limiting the validity of findings by overlooking these distinct aspects of perceived power.

Recognizing the conceptual disparity between personal and social power, there is a clear need for a tool that distinguishes between these two aspects. Our research addresses this gap by introducing a scale measuring individuals’ perceived ability to influence others and their perceived strength to resist external pressures. This scale enhances the understanding of power dynamics across various behaviors and augments the existing power literature.

Our first contribution lies in methodology. Prior research predominantly relies on measures of power that may not fully capture one’s perceived ability to control others, offering an incomplete picture of power effects. Although limited research on social versus personal power exists [18,21], a formally validated scale explicitly tapping into both aspects is lacking. The two-factor scale developed in our research fills this gap, providing a new method for researchers to explore both facets of power.

Our second contribution is theoretical. While not disputing prior research that conflates social and personal power, our framework offers a platform for exploring situations where the effects of these powers may diverge. To establish and validate these distinct roles, we conducted multiple studies. Study 1 focuses on establishing the orthogonality of the two power dimensions, showing that personal and social power can be manipulated independently. Study 2 highlights how personal power motivates proactive advice-seeking, whereas social power diminishes the likelihood of soliciting advice. Study 3 shows that social power amplifies negative reactions to service failures, while personal power does not. These results provide direct evidence that the two dimensions of power operate differently, thereby highlighting their distinctiveness. Lastly, in the General Discussion, we propose several avenues for future research and elucidate how this two-factor scale can spur further theoretical and empirical advancements in this realm.

## Theoretical background

At a broad level, power is defined as the ability to exert one’s will [18,22,23]. Research on power has largely focused on one’s ability to dictate the behavior of others due to an asymmetric control over resources in a social relationship [16,24,25]. However, scholars have moved beyond a unidimensional view of power, recognizing an important distinction between social and personal aspects of power [18,22]. More specifically, the concept of power has two dimensions [26]: (1) social power, or the power to control others’ outcomes, a form of influence [16], and (2) personal power, representing power over one’s own outcomes, related to autonomy [17].

Social power is defined as the relative capacity to change the behaviors and outcomes of other individuals [25]. Research supports the ubiquity of social power dynamics in diverse contexts, not only within hierarchical organizations where managers can direct their employees [21] but also in personal relationships where one might influence their spouse’s actions [27]. These dynamics extend to contexts like peer groups in colleges where social power can dictate behavior [28], emphasizing its broad relevance beyond work and marriage scenarios [29]. Having social power can be understood as possessing the ability to influence others and make them do things they may not do otherwise to satisfy a need for influence [30,31].

In contrast, personal power refers to the capacity to control one’s own outcomes, resist the influence of others and shape one’s own destiny [17]. As its core lies autonomy–the fundamental need for independence and self-governance [32,33]. This form of power enables individuals to act in accordance with their values and beliefs, even in the face of social conformity pressures. A sense of autonomy is a core innate psychological need [33], and its absence triggers negative emotions and compensatory behaviors [13]. When people experience a lack of personal power, they become motivated to regain control, associated with autonomy [34].

While personal power shares similarities with autonomy and independence, it is a distinct construct. Autonomy represents a fundamental psychological state characterized by self-endorsed actions and the experience of volition [33]. As an internal psychological process, autonomy reflects an individual’s capacity for self-governance and authentic decision-making, regardless of their relationships with others [35]. Personal power, however, encompasses elements that extend beyond this self-governance. It involves not only independent decision-making but also the active capacity to resist external influences and maintain control over one’s circumstances [17]. For example, a son might demonstrate high personal power by firmly refusing his parents’ pressure to become a doctor, yet ultimately choose a career in law that doesn’t align with his genuine interests, indicating low autonomy.

Understanding power through the dual lenses of influencing others (i.e., social power) and resisting influence (i.e., personal power) provides a comprehensive framework that highlights power as not solely about dominance over others but also about self-determination and control over one’s own life [21]. In today’s digital era, where individuals are immersed in digitally mediated spaces that expose them to constant social influence and external pressures [36], maintaining autonomy and resisting undue influence becomes increasingly challenging. As a result, personal power – the capacity to resist external influence and assert control over one’s decisions – has become increasingly significant.

Historically, power measures have primarily focused on social power, conceptualized as power over others. This trend is evident in the Personal Sense of Power Scale [20], which emphasizes an individual’s perceived capacity to influence others. Lammers et al. (2009) attempt to tap into various facets of power using single-item measures. While these measures have advanced our understanding of power, their emphasis on social power potentially restricts a comprehensive exploration of the construct. Existing measures often group personal power under the umbrella of social power or fail to capture it adequately. Yet, personal power, as a unique aspect, warrants equal attention to social power.

Moreover, measures targeting general power, such as Lammers et al.‘s (2009) and Anderson & Kilduff’s Trait Dominance measure (2009), aim to capture an overarching sense of power or dominant traits but often fail to account for the nuanced interplay between social and personal power. These measures may overlook how these dimensions differently affect behavior, particularly in contexts saturated with external influences.

These limitations suggest that while existing measures provide valuable insights, they are somewhat restricted in scope, primarily focusing on social power. For instance, the Personal Sense of Power scale [20] evaluates a global sense of power and whether others take one’s opinion or view into consideration, ignoring the fact that perception of power can also be understood as one’s perceived capacity to resist others’ influence. Moreover, these measures may even be ineffective for assessing perceived social power because it is possible to listen to someone else’s viewpoints while still acting contrary to their wishes.

Given these observations, there is a clear need for a scale that distinguishes between the two dimensions of power: social and personal. These dimensions are distinct and theoretically and practically divergent, suggesting they may engender different effects [18,21]. Thus, it may not be accurate to assert, “The effect of power is X,” when “The effect of social power is X, while the effect of personal power is Y,” may be more appropriate. By differentiating and validating a new scale that taps into both facets of power, we can enhance our understanding of power’s effects on behavior.

In the present research, we develop and validate a scale measuring personal and social power across multiple studies. We begin with exploratory factor analyses to identify the underlying structure and refine scale items, followed by confirmatory factor analysis to verify the two-factor structure. After establishing the scale’s reliability through internal consistency tests and a test-retest study, we demonstrate its nomological and discriminant validity by examining relationships with theoretically related psychological constructs. Finally, we showcase the scale’s practical utility through three experimental studies that demonstrate: (1) personal and social power can be orthogonally manipulated, (2) the two power dimensions differentially predict advice-seeking behavior, and (3) they have distinct effects on responses to service failures. Materials for all studies are accessible via the following link (https://doi.org/10.6084/m9.figshare.24045378).

## Scale development

The authors invited human participants to conduct behavioral studies for this research. Before conducting the studies, we’ve had an approval from Virginia Tech Institutional Review Board (IRB). The IRB approval number is 19–261. We’ve taken informed consent from our subjects before the studies.

The studies reported in this research article were approved by the Virginia Tech Institutional Review Board. Participants provided informed consent through an implied method. All participants viewed a research subject consent form before starting the survey, and consent was implied by the return of the completed questionnaire. The IRB waived the need for written documentation due to the minimal risk posed by the research. No minors were included in the study.

### Exploratory factor analysis

As an initial step [37], we reviewed the existing literature on social versus personal power and generated a pool of 20 items that represented each of the two domains. Among the items were 6 negatively-keyed items to reduce agreement acquiescence. Examples are as follows.

I can ignore others when I make my decisions.

Others have little to no say regarding what I do.

I have an ability to control others to get something I want.

I dictate what others do.

Given the number of items and expectation of high item communalities, we determined to use a sample size of approximately 200 in our exploratory factor analyses [38,39]. 201 US respondents whose primary language is English were recruited from MTurk (N =  201, ${\text{M}}_{\text{age}}$ =  35.74, ${\text{SD}}_{\text{age}}$ =  10.92, Female =  47.76%) using simple random sampling (described as Sample 1 in Table 1).

**Table 1:** Samples used in this research and reliability results.

*Sample*	*N*	*% Female*	*Mean Age*	*Mean Education* ^a^	*Mean Annual* *Household Income* *(In $1,000s)*	*Cronbach’s Alpha* ^b^
Sample 1	201	47.76	35.74	3.59	62.58	.81/.90
Sample 2	244	44.30	35.66	3.57	63.83	.84/.92
Sample 3	275	57.82	20.80	3.00	123.79	.81/.88
Sample 4	320	52.50	38.34	3.72	66.54	.86/.91
Sample 5	213	49.30	39.65	3.73	67.08	.85/.92
Sample 6	243	49.38	40.81	3.60	67.31	.84/.92
Sample 7	247	43.72	36.45	3.51	57.34	.86/.93
Sample 8	220	44.55	37.94	3.91	78.42	.86/.92
Sample 9	220	45.45	41.38	3.81	63.14	.86/.94
Sample 10	247	48.18	38.75	3.79	54.59	.89/.93
Sample 11	202	53.96	39.20	3.72	64.52	.87/.93
Sample 12	246	44.72	39.16	3.79	67.36	.88/.95

^a^1 =  “some high school”, 2 =  “high school graduate”, 3 =  “some college”, 4 =  “college graduate”, and 5 =  “masters/PhD graduate.”.

^b^The first number indicates Cronbach’s Alpha for personal power, the second for social power.

They were asked to rate each item on a seven-point Likert scale (1 =  “Strongly disagree,” and 7 =  “Strongly agree”). To prepare for factor analyses, we used SPSS to perform the Kaiser-Meyer-Olkin test of sampling adequacy [40] and Bartlett’s test of sphericity [41]. The result showed that the Kaiser-Meyer-Olkin measure of sampling adequacy was within an acceptable range (total matrix sampling adequacy = .90), and Bartlett’s test of sphericity was significant (*p* < .001), suggesting that the data was appropriate for factor analyses.

Next, conducted an exploratory factor analysis as part of the item-selection process. Specifically, we utilized oblique rotation using the R library *GPArotation* Version 2014.11-1 [42], because we assumed the latent variables are correlated to some extent, and that we will see similar results with Varimax rotation if the factors happen to be uncorrelated. The result of the parallel analysis suggested a three-factor structure rather than a two-factor structure as we expected. This structure arose due to all six negatively-keyed items loading distinctly onto a separate factor, appearing to represent the reverse coding method rather than a meaningful component of our construct. Following prior research [37,43], we attempted to resolve this problem by reverse coding some of the positively coded items and removing the original reverse coded items. Despite two attempts, the problem persisted. As the inclusion of the negatively-keyed items caused a systematic bias in the factor structure of our scale [44], we decided to drop all the negatively-keyed items from the scale. We acknowledge that there are benefits of having negatively-keyed items on a scale, such as reducing agreement bias [45]. However, including negatively worded items on a scale may be confusing for respondents due to reversals in item polarity. Moreover, research has shown that negatively-keyed items frequently form a separate method factor, as was the case in our samples, which does not appear to be meaningful [46]. Further, the presence of careless respondents, which is quite inevitable in most cases, can lead to a poor model fit [47].

Because of the complexities associated with the problems mentioned above, Brown and Marsh [48,49] recommended that scale developers consider including only either negatively-keyed items or positively-keyed items. Additionally, DeVellis (2016) noted that the disadvantages of the inclusion of negatively-worded items outweigh the benefits. Given this, we dropped all the negatively-keyed items. Also, we removed four other items that were either loading high on more than one factor or had loadings lower than.30 [50]. As a result, we utilized 10 positively-keyed items in our scale (i.e., five items for each type of power; see Table 2). However, in order to reduce agreement bias, we added bogus items as attention checks [51] and gave participants instructions warning them that the survey has attention-check questions [47].

**Table 2:** The scale items and factor loadings.

*Items*	*Personal Power*	*Social Power*	*Mean*	*SD*	*Communality*	*Item-Total Correlation*
Factor 1: Personal power						
1. Others have little to no say regarding what I do (P4).	.84		4.42	1.62	.70	.81
2. Others’ opinions do not stop me from how I would act or behave (P2).	.76		4.47	1.67	.58	.75
3. I have a feeling that I could freely choose to do whatever I want (P3).	.73		5.05	1.48	.54	.73
4. I have full control over what I do (P5).	.68		5.36	1.38	.45	.66
5. I can ignore others when I make my decisions (P1).	.59		4.49	1.72	.38	.61
Factor 2: Social power						
6. I dictate what others do (S4).		.87	2.70	1.52	.75	.86
7. People take orders from me (S3).		.86	3.08	1.64	.74	.86
8. I have an ability to control others to get something I want (S1).		.84	3.19	1.63	.72	.83
9. I can make others do things that they would not do otherwise (S5).		.84	2.99	1.67	.70	.83
10. Very often people adjust their behavior based on my opinions (S2).		.73	3.39	1.61	.53	.72

The final set of ten items incorporated in our scale is intentionally designed to capture the two distinct dimensions of power: social and personal. The domain of social power is represented by items explicitly conceived to encapsulate an individual’s perceived ability to modulate others’ behaviors. Items such as “I can make others do things that they would not do otherwise,” “I dictate what others do,” and “People take orders from me,” distinctively illustrate the capacity to influence others. Moreover, we have included items such as “I have an ability to control others to get something I want,” and “Very often people adjust their behavior based on my opinions,” to embody the subtler forms of social influence.

Regarding personal power, we aimed to encapsulate the ability to ignore and resist the influence of others. This is reflected in items such as “I can ignore others when I make my decisions,” and “Others’ opinions do not stop me from how I would act or behave,” illustrating an individual’s resistance to outside influence. We further explicate the idea of autonomy with items like “I have a feeling that I could freely choose to do whatever I want,” “Others have little to no say regarding what I do,” and “I have full control over what I do.” Thus, our scale offers a comprehensive measurement of personal power, delving deeper into the notion of autonomous action and decision-making, delineating the construct with precision missing from existing power scales.

With another group of 244 US respondents from MTurk (described as Sample 2 in Table 1; 7 participants were removed due to failure to pass our attention check items; the recruitment period started and ended on 16th May 2019), we conducted a principal axis factor analysis again on the new 10-item scale (N =  244, ${\text{M}}_{\text{age}}$ =  35.66, ${\text{SD}}_{\text{age}}$ =  10.36, Female =  44.30%). Both the parallel analysis and the scree plot suggested that two factors are sufficient. The final factor loadings of 10 items are listed in Table 2. The loadings of the items were above.50, and the two factors accounted for approximately 61.31% of the total variance (Personal power =  26.51%, Social power =  34.80%; See supporting information (S1 Table & S2 Table) for methodological details about the two initial EFA outcomes).

### Confirmatory factor analysis

**Fig 1:**
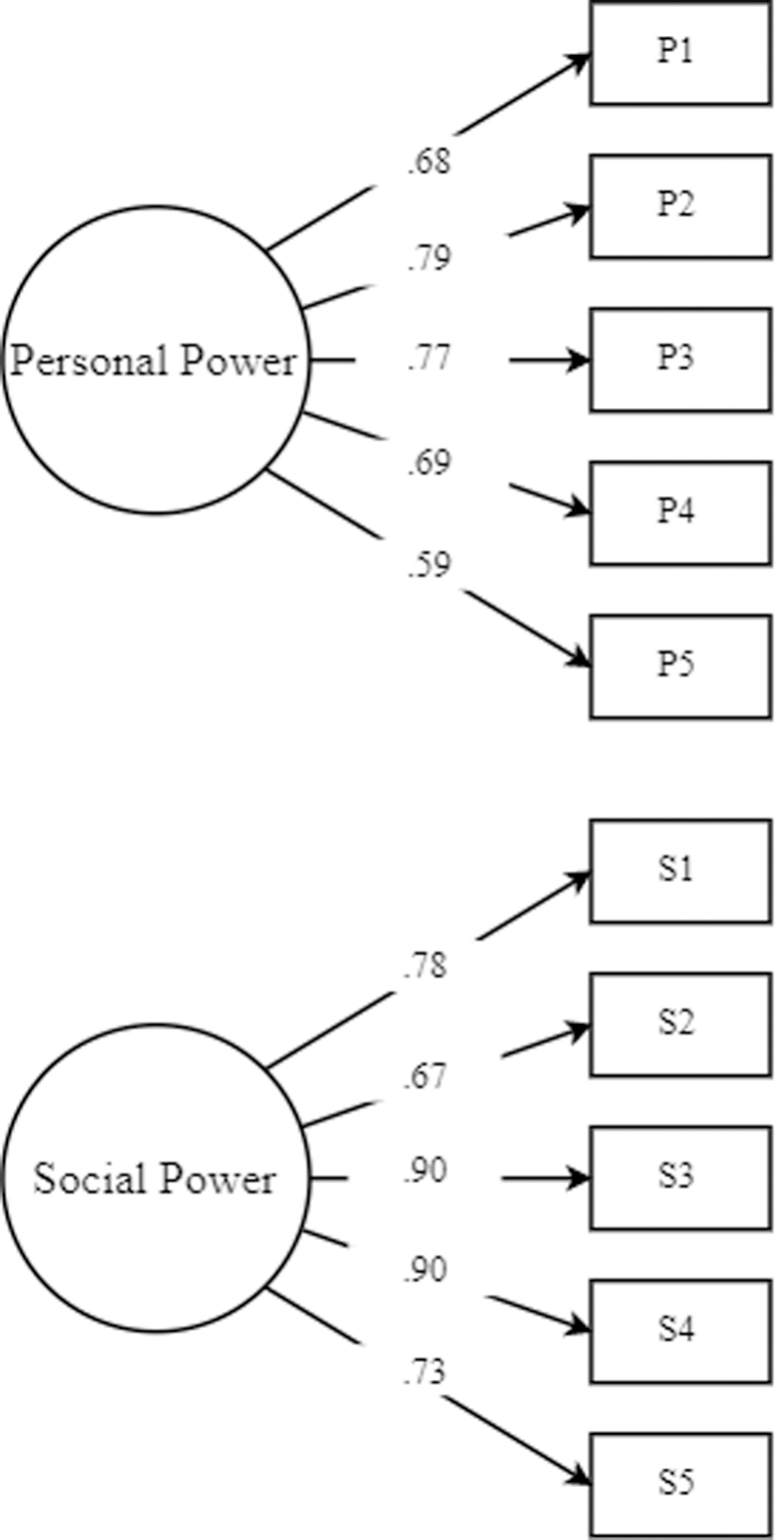
Confirmatory factor analysis results.

After the exploratory factor analysis, we recruited a group of undergraduate students at a large US university (described as Sample 3 in Table 1; N =  275, ${\text{M}}_{\text{age}}$ =  20.80, ${\text{SD}}_{\text{age}}$ =  1.15, Female =  57.82%) to conduct a confirmatory factor analysis. The recruitment period started and ended on 17th May 2019. Our final samples do not include respondents who failed to pass attention check items. We did not conduct other data cleaning steps, such as removing outliers. This rule was applied to all the studies reported in this article. We used a diagonally weighted least squares (DWLS) estimator in the R library *lavaan* Version 3.5.1, in which the estimator is called “WLSMV” [52]. The CFA data showed that the two-factor model fit the data well (${\text{x}}^{\text{2}}$ (34) =  81.07, CFI = .985, TLI = .980, RMSEA = .071, SRMR = .049). Those values were compared against the fit indices of the one-factor model. The fit increased significantly when the two-factor model was used ($\Delta {\text{x}}^{\text{2}}$ (1) =  326.74, *p* < .001). All the factor-loading estimates were significant (see Fig 1). Additionally, the two factors were estimated to be uncorrelated (r = .01, *p* = .811). However, the correlation between the two factors varied across the different samples, and we will address this further in the General Discussion.

To summarize, we developed the power scale of 10 items using exploratory factor analysis, and the two-factor model we hypothesized was confirmed through confirmatory factor analysis.

### Scale reliability

The internal consistency of the scale (i.e., Cronbach’s alpha) was assessed using the data collected for the exploratory factor analysis (Samples 1 and 2), the data for the CFA (Sample 3), the data for the test-retest reliability (Sample 4), and the data for nomological and discriminant validity (Samples 5, 6, 7, 8, and 9). The first study was started on 28th March 2019 and the last study ended on 17th May 2019. The results indicate that all of the Cronbach’s alphas were greater than.80, suggesting high internal consistency.

To assess the test-retest reliability of the scale, we administered the scale twice to 320 US respondents from MTurk (described as Sample 4 in Table 1; N =  320, ${\text{M}}_{\text{age}}$ =  38.34, ${\text{SD}}_{\text{age}}$ =  11.68, Female =  52.50%). with a one-week interval between the two administrations. The between-administration correlations showed high test-retest reliability (Personal Power = .77 (*p* < .001), Social Power = .75 (*p* < .001)).

### Validity analyses: Nomological and discriminant validity

Next, we demonstrate how our scale not only aligns with the theoretical expectations of the construct but also maintains its distinctiveness from other conceptually similar measures. For this purpose, we examined its correlations with the following scales: the Autonomy Scale [53], the Self-Construal Scale (SCS) [54], the Rosenberg Self-Esteem Scale [55], the General Self-Efficacy Scale [56], the Sense of Control Scale [57], the Self-Control Scale [58], the Personal Sense of Power Scale [20], and the Trait Dominance Scale [59]. These scales were selected on the basis of their potential theoretical connectedness with the scale we developed. Detailed descriptions of each measure are presented below. We will briefly explain how the scale we developed could be related to but conceptually distinct from these scales.

#### The autonomy scale.

This scale measures individual differences in autonomy, which consists of three subscales: (1) Sensitivity to others, (2) Capacity for managing new situations, and (3) Self-awareness [53]. This scale contains a total of 30 items. Example items include:

I often go deeply into other people’s feelings.

I need a lot of time to get accustomed to a new environment.

I often don’t know what my opinion is.

While the constructs of personal power and autonomy are correlated, they are not identical. Personal power, defined as an individual’s capacity to resist the influence of others, is a relational concept dependent on interpersonal dynamics. Conversely, autonomy is an internal state that denotes an individual’s ability for self-governance, irrespective of one’s power dynamics with others. For instance, a person may feel a low sense of autonomy due to a lack of self-awareness about personal goals, which is unrelated to their perceived ability to resist the will of others [35]. Therefore, while we hypothesize that personal power will be positively correlated with autonomy, the constructs are distinct and capture unique aspects of a person’s experiences.

The hypothesized relationship between social power and autonomy, although less direct, is impactful. Social power, defined as an individual’s capacity to influence others’ actions or decisions, often enhances an individual’s perceived autonomy indirectly. Those with high social power can often access more resources, wider networks, and exert more control over their social environment. These elements, in turn, can subtly boost their sense of self-governance or autonomy, by providing increased options and choices.

However, we acknowledge that high social power can sometimes be constrained by external factors, such as the expectations of others [18]. Hence, we propose that while social power contributes to autonomy, the correlation may not be as direct or robust as with personal power. Thus, we hypothesize a positive, albeit weaker, correlation between social power and autonomy.

#### SCS.

The 24-item Self-Construal Scale developed by Ted Singelis (1994) measures how people view themselves in relation to others. This scale is largely divided into two main subscales, each representing a distinct self-construal: an independent self-construal, emphasizing personal independence and individuality, and an interdependent self-construal, stressing connectedness and social relationship. Example items include:

I’d rather say “No” directly, than risk being misunderstood.

I have respect for the authority figures with whom I interact.

While there are expected correlations between the SCS scale and our power scale, particularly in the context of personal power, it’s critical to clarify the distinction between these constructs. For instance, we hypothesize that people with higher personal power will exhibit a stronger independent self-construal, as their ability to resist others’ influence might coincide with a more individualistic self-view. However, the relationship between social power and either independent or interdependent self-construal is less straightforward. Individuals with higher social power could potentially demonstrate a stronger interdependent self-construal due to their influential roles within their social networks. Alternatively, these individuals might show a more independent self-construal, given their distinctive position relative to others.

Regardless of these potential correlations, it is crucial to note that the constructs of self-construal and personal/social power are conceptually distinct. Self-construal pertains to the way individuals see their identity in relation to others, irrespective of their personal or social power. In contrast, our power scale focuses on the ability to influence or resist the influence of others. Although we hypothesize correlations, we underscore that these constructs each signify unique aspects of an individual’s social and personal experiences, providing a more nuanced understanding of their interplay.

#### The Rosenberg self-esteem scale.

This scale is a widely employed measure assessing global self-esteem [55]. Included are 10 items that pertain to self-worth and self-acceptance. Examples of these items include:

On the whole, I am satisfied with myself.

At times I think I am no good at all.

While we anticipate correlations between our power scales and the Rosenberg Self-Esteem Scale, the constructs these measures assess are not identical and we would like to clearly delineate them.

With regards to personal power, we hypothesize that it will show a positive correlation with self-esteem. The capacity to resist others’ unwelcome influences, a key aspect of personal power, may bolster one’s self-esteem in social contexts. However, the two constructs should not be considered identical. Self-esteem embodies an evaluative component of the self-concept, whereas personal power does not incorporate this self-evaluative aspect, focusing instead on the capacity to resist the influence of others.

As for social power, it could also exhibit a positive correlation with self-esteem, as being capable of controlling others can fortify one’s sense of self-worth. Nevertheless, we emphasize the critical distinction between social power and self-esteem. Social power is inherently relational, focusing on the extent to which one has control over others. In contrast, self-esteem is an intrapersonal measure, centered on the individual’s self-evaluation and perception of self-worth.

#### The general self-efficacy scale.

Chen and his colleagues developed this scale in order to assess how much people believe they can achieve their goals [56]. The scale consists of eight items, to which respondents indicate their level of agreement. Example items include:

I will be able to achieve most of the goals that I set for myself.

When facing difficult tasks, I am certain that I will accomplish them.

We hypothesize that there may be a positive correlation between both personal and social power and self-efficacy. People with high personal or social power often exhibit a heightened capacity for goal accomplishment, which may be mirrored in their self-efficacy.

However, it is crucial to maintain a clear conceptual distinction between self-efficacy and personal/social power. The notion of power relates to an individual’s perceived control in relation to others. More specifically, one’s ability to influence or resist influence from others. Self-efficacy, on the other hand, captures an individual’s confidence in their ability to achieve desired outcomes [60,61], focusing on the expectation of personal mastery and success. This belief in one’s capability is irrespective of the influences of others, thus signifying a distinct construct.

#### Sense of control.

Developed by Lachman and Weaver (1998), this scale examines a person’s sense of control over his or her life. It is divided into two subscales, personal mastery and perceived constraints. Example items include:

I can do just about anything I really set my mind to.

There is little I can do to change the important things in my life.

We hypothesize a positive correlation between both personal and social power and an individual’s sense of control. Personal and social power can often imply an enhanced degree of control, suggesting a possible relationship with the sense of control construct. Specifically, it may be that those with high personal power can perceive greater agency in directing their life path, enhancing their sense of control. Similarly, individuals with high social power, by guiding social interactions, may also perceive an increased sense of control.

Still, it is essential to highlight that these constructs, while potentially correlated, are fundamentally distinct. A sense of control is a broad belief in one’s ability to master, control, and shape their life [62]. In contrast, personal power specifically refers to one’s ability to resist others’ influences. Thus, an individual might confidently resist external influence but not necessarily feel mastery over certain aspects of life. Similarly, social power and a sense of control are conceptually distinct. A person may be able to exert control over others but lack the belief that they can effectively change their own life circumstances.

#### The self-control scale.

The Self-Control Scale [58] assesses individual differences in self-control, with a special focus on operational aspects. That is, it measures whether a person can override or change one’s inner responses. Example items include:

I’m good at resisting temptation.

People would say that I have iron self-discipline.

We hypothesize a positive relationship between personal power and self-control, given that both constructs share a theme of regulating one’s behaviors and responses. Personal power, as we defined it, concerns an individual’s ability to resist others’ influences. This necessitates a degree of self-control, as individuals must have the ability to regulate their own actions and not be swayed by others.

A similar proposition can be made for the relationship between social power and self-control. Specifically, those who hold social power have the capacity to influence others’ behaviors, which often requires strategic self-regulation. The effectiveness of social influence largely depends on one’s ability to control own actions and reactions in social contexts, thereby eliciting desired responses from others.

Despite the hypothesized correlations, these constructs reflect different nuances. That is, the constructs of personal and social power diverge from the ability to alter or control one’s inner responses. Central to our definition of personal (vs. social) power is the ability to assert control over oneself (vs. others), whereas the Self-Control Scale emphasizes an individual’s capacity to refrain from succumbing to undesired behaviors or impulses.

#### Personal sense of power.

This scale, devised by Anderson et al. (2012), measures one’s ability to influence others. Example items include:

I can get him/her/them to listen to what I say.

If I want to, I get to make the decisions.

My ideas and opinions are often ignored (R).

While our scale and the Personal Sense of Power scale may appear superficially similar, our scale is less ambiguous and more clearly taps into one’s perceived levels of power.

It is plausible that someone could score relatively high on this scale without necessarily having control over the behavior of others (i.e., social power) or having the capacity to resist the influence of others (i.e., personal power). For instance, an individual may perceive others to be receptive to their ideas without feeling able to shape those individuals’ actions or resist their influence (e.g., “Yes, I heard what you have to say, but I am still doing what I want”). Similarly, decision-making power may exist within certain parameters defined by others, indicating a susceptibility to external influence rather than personal power (e.g., “ordering” a group of children to eat ice cream).

These nuances suggest the distinctiveness of our scale in capturing the multi-dimensionality of power dynamics. We posit that although there may be a positive correlation between both of our subscales and the Personal Sense of Power scale, they represent unique aspects of power perception. Therefore, we hypothesize that our scale will show discriminant validity from the Personal Sense of Power scale.

#### Trait dominance.

Three items from the Revised Interpersonal Adjective Scales (IAS-R) [59] measure individuals’ tendency to behave in assertive, forceful, and self-assured ways. Example items include:

Please rate how accurately the word ‘dominant’ describes you as a person.

Please rate how accurately the word ‘assertive’ describes you as a person.

Please rate how accurately the word ‘forceful’ describes you as a person.

Trait dominance is hypothesized to show a positive relationship with both personal and social power. Trait dominance, characterized by assertiveness and self-assuredness, mirrors behaviors common in power dynamics, suggesting potential correlations with personal and social power. Specifically, individuals high in social power often exert considerable influence over their environment, a characteristic mirrored in the assertiveness and forcefulness typical of trait dominance. Personal power, on the other hand, is also anticipated to share a positive correlation with trait dominance as the ability to resist external influences, a central aspect of personal power, aligns with the self-assuredness and assertiveness embodied in dominant individuals.

However, despite these expected correlations, it’s essential to distinguish these constructs. Trait dominance signifies persistent behavioral patterns across varying situations, while personal and social power are relational constructs, relating specifically to the dynamics of influence within personal and societal contexts. One can display trait dominance while lacking personal or social power and vice versa, emphasizing the distinct nature of these constructs despite potential overlap. Maintaining this distinction is crucial to ensure discriminant validity.

We report the results of the discriminant analysis in Table 3. Five studies were conducted to examine the discriminant validity of our scale (described as Sample 5, 6, 7, 8, and 9 in Table 1). As indicated in Table 3, our scales for personal and social power demonstrate little to no correlation, or only weak correlations, with many of the considered variables ($\left|\text{r}\right|$ < .2), reinforcing the discriminant validity of our measures [37]. On the other hand, meaningful and significant correlations with constructs that were hypothesized to be related reflect our measures’ nomological validity, aligning with our theoretical explanations.

**Table 3:** Correlations of the power scale with other relevant scales.

	*Sample Used*	*Mean*	*SD*	*Cronbach’s Alpha*	*Personal Power*	*Social Power*
Autonomy	Sample 5	4.15	.74	.87	.462***	.126*
Interdependent Self-Construal	Sample 5	4.71	.93	.83	-.158**	-.076
Independent Self-Construal	Sample 5	5.14	.93	.83	.501***	.151**
Self-Esteem	Sample 6	5.16	1.35	.94	.358***	-.070
Self-Efficacy	Sample 6	3.81	.75	.93	.519***	.136**
Sense of Control	Sample 7	5.14	1.29	.94	.392***	-.099
Self-Control	Sample 7	3.45	.78	.89	.151**	-.167***
Personal Sense of Power	Sample 8	4.36	1.05	.77	.274***	.260*
Trait Dominance	Sample 9	3.49	1.52	.86	.271***	.654***

*p < .10,

**p < .05,

***p < .01

It is noteworthy to discuss the variables that showed a correlation with an absolute value between.4 and.6, as coefficients in this range are moderate [37,63]. Personal power was positively correlated with Autonomy, Independent Self-Construal, and Self-efficacy. Social power was positively correlated with Trait Dominance. As we discussed in detail above, it makes theoretical sense that our scale is correlated with these constructs, as perceived power should influence these variables. However, the fact that the correlations are within the moderate range is evidence that personal and social power are conceptually distinct from these constructs [63].

One may wonder why the correlation between social power and the Personal Sense of Power scale exhibited a relatively low correlation. The reason for this can be traced back to the fundamental differences in the operationalization of power in these two scales. As highlighted in our earlier discussions, the ability to directly control or dictate others’ behaviors, which is central to our social power scale, is distinct from the ability to merely have one’s views or opinions considered, which is the focal point of Anderson et al. (2012)’s scale. Therefore, the conceptual divergence between our social power construct and Anderson et al. (2012)’s Personal Sense of Power is expected. Instead, this underscores the distinct focus of our scale on unambiguous perceptions of power. In addition, our analysis revealed a quite interesting relationship between power and self-control. More specifically, personal power was positively related to self-control, while social power was negatively related. We discuss this finding further in the General Discussion.

To further assess discriminant validity beyond simple correlations, we conducted an additional test using the Heterotrait-Monotrait (HTMT) ratio [64]. Monte Carlo simulation studies have demonstrated that the HTMT method achieves higher specificity and sensitivity compared to other techniques [64,65]. Use of HTMT criterion involves comparing HTMT values to a predefined threshold, with 0.85 commonly recommended [66]. Values exceeding this threshold indicate a lack of discriminant validity. Our analysis revealed that all constructs have HTMT values below 0.85, confirming that our power scale is distinct from related constructs. We report the results of the HTMT analysis in Table 4.

**Table 4:** Heterotrait-monotrait ratio results.

	*Personal Power*	*Social Power*
**Sample 5**	–	–
Autonomy	.214	.112
Interdependent self-construal	.555	.044
Independent self-construal	.500	.624
**Sample 6**	–	–
Self-esteem	.613	.797
Self-efficacy	.157	.064
**Sample 7**	–	–
Sense of control	.274	.603
Self-control	.105	.104
**Sample 8**	–	–
Personal sense of power	.441	.793
**Sample 9**	–	–
Trait dominance	.298	.740

Note: HTMT stands for Heterotrait-Monotrait ratio. The numbers represent HTMT values, and values close to 1 indicate a lack of discriminant validity.

We have affirmed that our scale is a robust instrument and have successfully demonstrated that our conceptualization of personal/social power is not only distinct from related constructs but also consistently correlates with theoretically related variables, thereby confirming its nomological validity. Next, we present the results of three studies that further demonstrate the value of our scale. Specifically, we show that personal and social power can be manipulated orthogonally, and that personal and social power can result in divergent effects. In all studies, we aimed for a sample size of approximately 200, as that gives us 80% power to detect a correlation with an effect size of.20.

## Study 1

Having established the psychometric properties of our scale, we next demonstrate its construct validity and versatility in capturing both trait and state experiences of power. While power has traditionally been studied as a stable individual difference variable, research has shown that power is also a mental construct that can be situationally primed and activated [67]. Building on this understanding, we examine whether our scale can capture situational variations in feelings of personal and social power.

We predict that properly designed recall tasks should be able to selectively influence these distinct power dimensions, even though prior research has often conflated them in experimental manipulations. To test this, we conducted a 2 X 2 between-subjects experiment where participants recalled situations of high/low personal power and high/low social power, then completed our power scale.

### Method

A total of 252 US-based MTurk respondents took part in this study, which started and ended on 5th May 2023. After removing five participants who failed our attention check items, we had a final sample of 247 participants (described as Sample 10 in Table 1; N =  247, ${\text{M}}_{\text{age}}$ =  38.75, ${\text{SD}}_{\text{age}}$ =  11.54, Female =  48.18%). All data deletion was done on *a priori* grounds and data were not analyzed prior to these deletions. Participants engaged in two separate recall tasks, remembering situations where they felt high or low levels of personal and social power (see supporting information for the specific instructions used in Study 1). The order of task presentation was counterbalanced*,* resulting in a 2 (personal power: low vs. high) X 2 (social power: low vs. high) between-subjects design. After the recall tasks, participants completed our power scale (${\text{α}}_{\text{personal}}=$.89; ${\text{α}}_{\text{social}}=$.93) and standard demographic questions.

### Results

We performed two separate regression analyses. In the first, the personal power subscale served as the dependent variable, with the personal and social power manipulations and their interaction as independent variables. This analysis revealed a significant main effect of the personal power manipulation on the personal power subscale (β = .41; t (243) =  5.52; *p* < .001), while the main effect of the social power manipulation (β = .08; t (243) =  1.21; *p* = .226) and the interaction were not significant (β = .09; t (243) = .13; *p* = .900).

**Fig 2:**
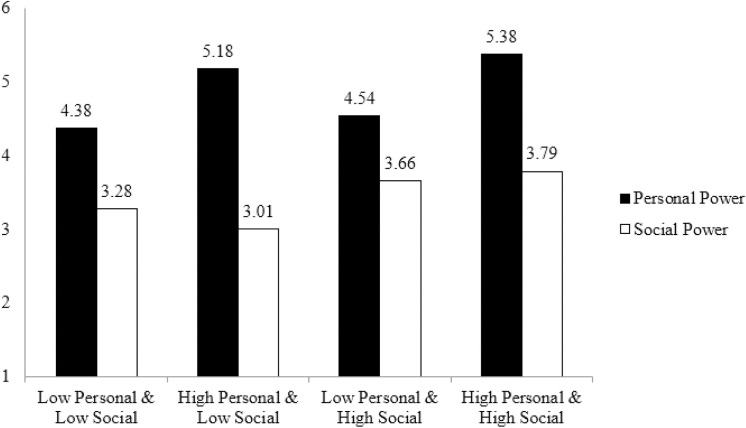
Personal and social power can be orthogonally manipulated.

When the social power subscale served as the dependent variable, we observed a significant main effect of the social power manipulation on the social power subscale (β = .27; t (243) =  3.44; *p* < .001). The main effect of the personal power manipulation (β =  -.04; t (243) = .43; *p* = .664) and the interaction were not significant (β = .10; t (243) =  1.22; *p* = .225). Please see Fig 2 for detailed information.

These results corroborate that personal and social power can be orthogonally manipulated, lending further support to the construct validity of our scale. Next, we will utilize our scale to demonstrate that there may be situations in which personal and social power effects diverge.

## Study 2

In the advice taking literature, numerous studies have revealed that people tend to discount advice from others [68]. That is, people underweight advice from others and over-weight their own opinions [69]. This tendency is particularly likely when a decision-maker feels optimistic about his/her decision, feels control over the decision, and feels confident about his/her ability in the decision [70]. As such, we hypothesize that power will influence the tendency to follow advice. However, we also posit that the direction of the effect depends on the type of power (i.e., personal vs. social).

Individuals high in social power are habituated to commanding and influencing others, which can foster an inflated self-perception of their knowledge and abilities [71]. This, in conjunction with the optimism emanating from the frequent exercise of power over others [19], could lead these individuals to undervalue the advice of others, decreasing the propensity to seek advice.

Conversely, those high in personal power are distinguished by their autonomy and ability to resist unwanted influences [26,72]. It does not inherently include perceptions of superiority or dismissiveness towards others’ opinions or competencies. Consequently, we hypothesize that individuals high in personal power are likely to be open to soliciting advice, using their autonomy as a means of discerning the utility of such advice without diminishing their control over decisions. That is, they can confidently choose to consider others’ inputs without feeling threatened or diminished in their power status.

## Method

Nine individuals failed to pass our attention check items, making our final sample 202 US participants from MTurk (described as Sample 11 in Table 1; N =  202, ${\text{M}}_{\text{age}}$ =  39.20, ${\text{SD}}_{\text{age}}$ =  12.16, Female =  54.0%). The recruitment period started and ended on 17th Sep 2019. First, participants completed the 10-item power scale we developed (${\text{α}}_{\text{personal}}=$.87; ${\text{α}}_{\text{social}}=$.93). After completing the scale, participants then imagined a situation in which they may consult others for their opinion [73]:

*You are assigned an important project to create a photo essay. The project requires using a photo album software package. After browsing on the Internet for a while, one software package caught your eye. The price of the software package is within your budget. Before making your decision, you found an online discussion forum for photo album software packages.*

After reading the scenario, participants responded to the following items: (1) “Given this situation, how likely would you be to consult online reviews given by other consumers (1 = Not at all likely, 7 = Very likely)?”, (2) “Given this situation, how useful do you think online reviews given by other consumers would be (1 = Not at all useful, 7 = Very useful)?”, and (3) “Given this situation, how relevant do you think online reviews would be for your decision (1 = Not at all relevant, 7 = Very relevant)?” These answers were averaged (*α* = .88) and served as our dependent variable. Finally, participants answered standard demographic questions.

### Results

We performed a regression analysis in which personal power, social power, and their interaction term served as independent variables, and the tendency to seek advice served as the dependent variable. The scores for both personal and social power were mean-centered before the analysis. The interactive effect of social and personal power on one’s tendency to seek advice was not significant (β = .06, t (198) =  1.39; *p* = .166), but the effects of personal power (β = .13, t (198) =  2.08; *p* = .039) and social power (β =  -.12, t (198) = –2.49; *p* = .014) were significant, indicating that the result was supportive of our prediction.

The results of this study suggest that as individuals experience higher levels of personal (social) power, they will be more (less) likely to seek advice from others. We think this is particularly interesting given that prior research suggests that there should only be a negative main effect of power [70]. We discuss this further in the General Discussion. Next, we examine the effects of personal and social power in the context of a service failure.

## Study 3

People often face negative consumption experiences, which may lead to complaints [74]. The topic of responses to service failure has attracted increasing attention from researchers [75–77]. We propose that power can have a differential impact on one’s response to service failure depending on whether one’s power is personal or social. We hypothesize a positive relationship between social power and the tendency to react negatively to service failures. However, there should be less of a relationship between one’s level of personal power and one’s reaction to a service failure. Service failure can be interpreted as a failure to control others. Given that social power taps into the ability to control others, people high in social power may consider service failure as a threat to their ability to control others, resulting in more negative responses [16]. In contrast, we posit that one’s level of personal power should have less impact on how individuals react to service failures. The notion of personal power is conceptualized as one’s ability to ignore or resist others’ influences, and a service failure does not represent a threat to this ability.

We also incorporated the Personal Sense of Power Scale into our study. This scale, developed by Anderson and colleagues (2012), primarily gauges an individual’s perceived influence over others, and has been extensively used in prior research. We included this additional scale for two reasons. First, we wanted to provide a benchmark against which we could evaluate the predictive ability of our newly constructed power scale. Second, the inclusion of this scale offers additional insights into how variations in measures of power can impact how researchers interpret the effects of power on behavior. Given that our scale more directly captures perceptions of personal and social power versus the Personal Sense of Power Scale, their simultaneous inclusion allows for a richer and more nuanced analysis of the role of perceived power in individuals’ responses to service failures.

### Method

A total of 251 US-based MTurk respondents participated in this study. The recruitment period started and ended on 7th May 2023. After eliminating five participants due to failed attention checks, we had a final sample size of 246 participants (described as Sample 12 in Table 1; N =  246, ${\text{M}}_{\text{age}}$ =  39.16, ${\text{SD}}_{\text{age}}$ =  9.94, Female =  44.72%). Participants first completed our power scale (${\text{α}}_{\text{personal}}=$.88; ${\text{α}}_{\text{social}}=$.95), then the Personal Sense of Power scale [20] (α = .80), then read a hypothetical service failure scenario [76]:

*Recently, you are looking for a nice restaurant to celebrate your birthday with your family. You ask Chris, an owner of Seaside Restaurant, whether you can reserve a table next week. You request a table with a sea view because you want to celebrate your birthday with your family.*

*Chris tells you that it is peak season, but he will try his best to arrange the table for you. Based on your past experience with Chris, you think that he will have the table for you next week. You agree with his arrangement. One week later, you go to the restaurant with your family and ask Chris to put you at a sea-view table. However, Chris tells you that all the sea-view tables are already taken. In this case, you cannot enjoy the sea view when having your birthday dinner with your family.*

After they read the scenario, participants were asked to respond to what extent they agree with the following items: (1) “I am dissatisfied with the service (1 = Strongly disagree, 7 = Strongly agree)”, (2) “I am unhappy with the service (1 = Strongly disagree, 7 = Strongly agree)”, and (3) “I am not pleased with what Chris has done (1 = Strongly disagree, 7 = Strongly agree)”. The average of these items served as the dependent variable (α = .97). Finally, participants answered standard demographic questions.

### Results

We conducted a regression analysis in which personal power, social power, and their interaction term served as independent variables, and the response to service failure served as the dependent variable. The scores for both personal and social power were mean-centered before the analysis. The interactive effect of social and personal power on one’s response to service failure was not significant (β = .06, t (242) =  1.01; *p* = .315). Most importantly, the effect of social power (β = .15, t (242) =  1.95; *p* = .05) was significant, while the effect of personal power was insignificant (β = .01, t (242) = .11; *p* = .916). The results of this study suggest that as social power increases, individuals will react more negatively to service failures. However, one’s level of personal power does not influence how negatively they respond to service failures.

We also examined the association between the Personal Sense of Power and the response to service failure. The Personal Sense of Power had significant correlations with our personal power (r = .32; *p* < .01) and social power (r = .27; *p* < .01) subscales. More importantly, our analysis revealed a non-significant relationship between the Personal Sense of Power and the response to service failure (β = .02, t (244) = .14, *p* = .887). This finding further underscores the differential predictive ability of our social power subscale compared to the Personal Sense of Power Scale. Despite the theoretical relevance of the Personal Sense of Power, its utility in predicting responses to service failure, as demonstrated in our study, appears limited. This bolsters our argument for the superior predictive validity of our power scale in the context of service failures, providing additional empirical support for its adoption in research settings.

## General discussion

In this research, we highlight the importance of making a distinction between social and personal power. Despite this importance in research, researchers often overlook that the notion of power can be conceptualized as having two different dimensions. To address this issue, we develop a 10-item scale to measure one’s perceived social and personal power, providing a tool that can be applied across diverse fields, including social psychology, communication, marketing, and economics. We demonstrate the reliability and validity of the scale and also show its predictive ability in multiple studies. Next, we discuss the implications of this research and directions for future research.

Although we conceptualized personal and social power as two distinct constructs, both are intrinsically linked through their foundational emphasis on influence. While personal power manifests as influence over one’s internal actions and decisions, social power embodies the ability to exert influence over the behaviors and choices of others. Despite their operational differences—whether internal or external—both forms emanate from a deep-seated human need for control and authority. In many scenarios, these powers may interplay, with robust personal power augmenting social influence and vice versa.

Supporting this conceptual understanding, the result from Sample 2 demonstrates a significant correlation between personal and social power (r = .16, *p* = .012). However, we also found that the correlation varies across samples (Sample 3: r = .01, *p* = .811; Sample 4: r = .01, *p* = .840; Sample 5: r = .10, *p* = .155; Sample 6: r = .18, *p* = .006; Sample 7: r = .11, *p* = .078; Sample 8: r = .39, *p* < .001; Sample 9: r = .24, *p* < .001; Sample 10: r = .18, *p* = .004; Sample 11: r = .16, *p* = .025; Sample 12: r = .30, *p* < .001), suggesting that we need a bigger sample size to estimate the relationship between the two constructs. In order to estimate the relationship between personal and social power, we collapsed the data from Sample 2 to Sample 12 except for Sample 10 in which we directly manipulated both personal and social power, resulting in a sample size of 2,430. With this dataset, we calculated the correlation between the two constructs and found a significant association between one’s feelings of personal power and social power (r = .17, *p* < .001). This result indicates that while personal and social power are conceptually distinct, they both draw from the overarching construct of power.

However, while both dimensions reflect aspects of power, the modest correlation and our findings of divergent effects across studies suggest value in examining personal and social power separately. Given that these dimensions can predict different outcomes, as demonstrated in our studies, researchers may benefit from analyzing them independently to capture potentially unique effects. At the same time, studying overall power remains valuable, particularly when examining broad power-related phenomena. We encourage researchers to consider their specific research questions when deciding whether to examine power as a unified construct or to investigate its personal and social dimensions separately.

The present research offers a new tool for estimating how power influences behavior by making a distinction between social and personal power. As we discussed earlier, researchers often confound social and personal power when they manipulate power. As a result, sometimes mixed results emerge regarding a seemingly identical phenomenon. For instance, as illustrated by Lammers and colleagues (2009), studies that found a positive effect of power on stereotyping usually manipulate power in independent settings, which is known to be associated with personal power. In contrast, studies showing a negative effect of power on stereotyping manipulated power in a way that participants can feel some sense of responsibility for others, which might be more associated with social power. Because of this potential confound, it was only after the researchers distinguished between the two types of power were it possible to clearly determine the effects of power on stereotyping.

In this research, we tested whether individuals’ tendency to seek advice varies as a function of the type of power experienced (i.e., personal vs. social power). This finding is interesting because prior research has shown that subjective feelings of power cause individuals to discount others’ advice [70]. One might question whether what we found is consistent with this research. We believe that our findings do not necessarily conflict with Tost and colleagues’ (2012) findings because there are differences between the studies conducted by Tost and colleagues (2012) and ours. In their studies, participants had the chance to make use of unsolicited advice. Specifically, participants saw pictures of three different individuals and estimated each person’s weight. After reporting their initial estimates, they had a chance to adjust their initial estimates while viewing another participant’s estimates about the same people. Thus, they investigated the effects of power on the use of unsolicited advice. In our study, however, we merely asked participants whether they were willing to consult online reviews before deciding. That is, we focused on whether different types of power can have diverging effects on one’s tendency to seek advice in the first place. Moreover, participants in Tost and colleagues’ (2012) studies may have motivations that are different from those in our study. Whereas respondents in their studies were motivated to be accurate and to prove their competence, participants in our study were more likely to be interested in choosing a reasonable product. Because of this difference, seeking advice can be thought to signal different things. In addition to these differences, Tost and colleagues (2012) also conflate personal and social power such that they manipulate social power in the high-power condition and personal power in the low power condition. Future research should address these issues to provide a better understanding of how power affects the tendency to seek advice.

In our studies, we show that our scale can predict the tendency to seek advice and the severity of negative reaction to a service failure, and that personal and social power can lead to very different results. Although these findings are correlational, we believe that they can stimulate future research. Might our scale predict other divergent outcomes in terms of personal versus social power? We believe that it very well may. One interesting finding we report in Table 3 is that one’s self-control can be differentially influenced by which types of power is activated. This finding is interesting not only because the results were significant in different directions, but because it is suggestive of the potential of our new scale as an effective theory testing tool. For instance, different theories have predicted different outcomes regarding how power might affect self-control. Some theories suggest that power may lead to disinhibition and poor decision making, as power leads to greater risk-taking [19], illusory control [78], and reward sensitivity [16,79]. Conversely, it is also possible that feeling powerful may help power holders to inhibit impulses [80], leading to higher self-control. Indeed, Joshi and Fast (2013) tested the effect of power on temporal discounting and found that enhanced power leads to reduced temporal discounting via increased self-continuity. However, other researchers cast doubt on this result by failing to replicate the result [81]. Given our correlational findings, perhaps the key to resolving this puzzle lies in examining the problem from the lens of personal versus social power. For instance, people high in personal are used to controlling their own outcomes. Thus, for these individuals, the concept of “control” is naturally connected to the concept of “self”. Conversely, those high in social power are accustomed to controlling others, meaning that for these individuals, the concept of “control” is not as connected to the concept of “self”. Future research could formally test these hypotheses, providing causal evidence to buttress our correlational findings.

Despite its contributions, the research has limitations. The scale’s development and validation rely on cross-sectional data from American respondents, necessitating future investigations into its applicability across diverse cultures. Additionally, our validation studies involve fictitious scenarios, urging the need for consequential studies to enhance the scale’s robustness.

To summarize, researchers have often overlooked the distinction between personal and social power when examining the effect of power [24]. We propose and develop a new scale for measuring the two separate facets of power, providing a powerful tool for theory building. We show that our new scale can predict a number of behaviors, and we expect that this scale can be helpful for theory testing when theories regarding power make divergent predictions.

## Supporting information

S1 Table Exploratory factor analysis (sample 1).
(DOCX)

S2 Table Exploratory factor analysis (sample 2).
(DOCX)
