# Supplementary material for: A two-factor scale of perceived power
Source: PLoS One. 2025 Feb 28;20(2):e0319412. doi: 10.1371/journal.pone.0319412 (PMC11870369; doi:10.1371/journal.pone.0319412)
Supplement: S1 Table — (DOCX) [file pone.0319412.s001.docx]

**S1 Table. Exploratory factor analysis (sample 1)**

| Items | Factor | | | Dimension |
| --- | --- | --- | --- | --- |
|  | 1 | 2 | 3 |  |
| P1 | .41 | -.45 |  | Personal Power |
| P3 | .73 |  |  |  |
| P4 | .76 |  |  |  |
| P5 | .63 |  |  |  |
| P10 | .70 |  |  |  |
| S1 |  | .85 |  | Social Power |
| S2 |  | .86 |  |  |
| S3 |  | .83 |  |  |
| S4 |  | .85 |  |  |
| S5 |  | .83 |  |  |
| S7 |  | .87 |  |  |
| S8 |  | .79 |  |  |
| S10 |  | .71 |  |  |
| P2 |  |  | .65 | Reverse-Coded |
| P6 |  |  | .85 |  |
| P7 |  |  | .81 |  |
| P8 |  |  | .59 |  |
| P9 |  |  | .64 |  |
| S6 |  |  | .76 |  |

*Notes*. Extraction method; principal axis factoring; Rotation method; Oblimin rotation. Loadings larger than .30 are reported.

**Appendix.**

Methodological details (EFA, sample 1)

Participants

201 US participants ($\text{M}_{\text{age}}$ = 35.74, $\text{SD}_{\text{age}}$ = 10.92, Female = 47.76%) recruited through MTurk.

Online Survey Design

Survey flow:

1. Participants read a brief instruction about the survey.
2. After a brief instruction, participants were presented with two blocks of candidate scale items (20 items total; 10 items assigned to each block).
3. Lastly, participants were asked to answer several demographic questions (age, gender, yearly household income, level of education).

Measures

“On the following screens, please read carefully and answer honestly.”

(1 = Strongly disagree; 7 = Strongly agree)

Candidate items for personal power:

P1. I avoid acting in accordance with the existing rules or norms.

P2. When making decisions, I feel constrained by others’ opinions (R).

P3. I can ignore others when I make my decisions.

P4. Others’ opinions do not stop me from how I would act or behave.

P5. I have a feeling that I could freely choose to do whatever I want.

P6. Others dictate what I do (R).

P7. Others play a big role in determining my actions (R).

P8. I take orders from others (R).

P9. When completing tasks, I have to think of what others would want me to do (R).

P10. Others have little to no say regarding what I do.

Candidate items for social power:

S1. I have an ability to control others to get something I want.

S2. I can make others to do things that they would not do otherwise.

S3. I have enough control over others’ tasks.

S4. I can influence others’ thoughts or behavior.

S5. Very often people adjust their behavior based on my opinions.

S6. My ideas and opinions are often ignored (R).

S7. People take orders from me.

S8. People feel as though they must listen to what I have to say.

S9. I dictate what others do.

S10. Other people must think of what I want when completing tasks.

Study Analysis

Analysis approach:

In our factor analysis, we opted for an oblique rotation using the R library *GPArotation* (Bernaards et al., 2015). This decision was predicated on our theoretical expectation that the latent variables under consideration are likely interrelated. Oblique rotations allow for the factors to correlate, often unveiling clearer, more meaningful factor structures especially when there's theoretical justification to expect such correlations (Netemeyer, Bearden, & Sharma, 2003).

Number of factors to retain:

The Kaiser-Meyer-Olkin measure verified the sampling adequacy for the analysis (total matrix sampling adequacy = .90), and Bartlett’s test of sphericity was significant (*p* < .001), suggesting that the data was appropriate for factor analyses. The principal axis factor analysis with a cut-off point of .30 and the Kaiser’s criterion of eigenvalues greater than 1 yielded a three-factor solution as the best fit for the data, accounting for 61.92% of the variance. As shown in the table, except for the negatively-keyed items, the rest of the items for personal and social power distinctly loaded onto two separate factors. The personal power items contributed to a factor explaining 31.52% of the variance, while the social power items constitute another factor, accounting for 18.31% of the variance. All negatively-keyed items clustered together to form a distinct factor, responsible for 12.09% of the variance.

It is worth noting that there’s an absence of a universally endorsed criterion dictating the exact number of factors to extract, as highlighted by Netemeyer et al. (2003). In such scenarios, it’s often advisable to employ multiple criteria in exploratory factor analysis to inform such decisions. Furthermore, prior theoretical understanding and intuitive reasoning should play pivotal roles in guiding the determination of factor retention.

Notably, it has been observed in previous literature that negatively-keyed items tend to converge into a distinct method factor, which was consistent with our findings. Also, this separate factor lacks substantive meaning and extant theoretical literature on power doesn’t seem to propose a three-factor structure akin to our findings.
