# Supplementary material for: A two-factor scale of perceived power
Source: PLoS One. 2025 Feb 28;20(2):e0319412. doi: 10.1371/journal.pone.0319412 (PMC11870369; doi:10.1371/journal.pone.0319412)
Supplement: S2 Table — (DOCX) [file pone.0319412.s002.docx]

**S2 Table. Exploratory factor analysis (sample 2)**

| Items | Factor | | | Dimension |
| --- | --- | --- | --- | --- |
|  | 1 | 2 | 3 |  |
| P4 | .84 |  |  | Personal Power |
| P2 | .77 |  |  |  |
| P3 | .74 |  |  |  |
| P5 | .68 |  |  |  |
| P1 | .59 |  |  |  |
| S4 |  | .87 |  | Social Power |
| S3 |  | .86 |  |  |
| S1 |  | .84 |  |  |
| S5 |  | .84 |  |  |
| S2 |  | .73 |  |  |

*Notes*. Extraction method; principal axis factoring; Rotation method; Oblimin rotation. Loadings larger than .30 are reported.

**Appendix.**

Methodological details (EFA, sample 2)

Participants

244 US participants ($\text{M}_{\text{age}}$ = 35.66, $\text{SD}_{\text{age}}$ = 10.36, Female = 44.30%) recruited through MTurk.

Online Survey Design

Survey flow:

1. Participants read a brief instruction about the survey.
2. After a brief instruction, participants were presented with two blocks of candidate scale items (10 items total; five items assigned to each block).
3. Lastly, participants were asked to answer several demographic questions (age, gender, yearly household income, level of education).

Measures

“On the following screens, please read carefully and answer honestly.”

1. = Strongly disagree; 7 = Strongly agree)

Candidate items for personal power:

P1. I can ignore others when I make my decisions.

P2. Others’ opinions do not stop me from how I would act or behave.

P3. I have a feeling that I could freely choose to do whatever I want.

P4. Others have little to no say regarding what I do.

P5. I have full control over what I do.

Candidate items for social power:

S1. I have an ability to control others to get something I want.

S2. Very often people adjust their behavior based on my opinions.

S3. People take orders from me.

S4. I dictate what others do.

S5.I can make others do things that they would not do otherwise.

Study Analysis

The Kaiser-Meyer-Olkin measure verified the sampling adequacy for the analysis (total matrix sampling adequacy = .84), and Bartlett’s test of sphericity was significant (*p* < .001), suggesting that the data was appropriate for factor analyses. The principal axis factor analysis with a cut-off point of .30 and the Kaiser’s criterion of eigenvalues greater than 1 yielded a two-factor solution as the best fit for the data, accounting for 61.31% of the variance. As shown in the table, the items for personal and social power distinctly loaded onto two separate factors. The personal power items contributed to a factor explaining 26.51% of the variance, while the social power items constitute another factor, accounting for 34.80% of the variance.

Instructions used in Study 1

| *Condition* | *Instructions* |
| --- | --- |
| Low Personal Power | Recall a situation in which you felt as though you did not have the ability to ignore and resist the influence of others.  In this situation, you couldn't do what you wanted and needed to follow orders or suggestions from others.  Please briefly write 2 sentences describing this situation. |
| High Personal Power | Recall a situation in which you felt as though you had a strong ability to ignore and resist the influence of others.  In this situation, you did what you wanted and ignored orders or suggestions from others.  Please briefly write 2 sentences describing this situation. |
| Low Social Power | Recall a situation in which you felt as though you did not have the ability to influence what others do or how they behave.  In this situation, you told others what to do and they did not follow your orders.  Please briefly write 2 sentences describing this situation. |
| High Social Power | Recall a situation in which you felt as though you had a strong ability to influence what others do or how they behave.  In this situation, you told others what to do and they followed your orders.  Please briefly write 2 sentences describing this situation. |
